# Frequency of fibrosis in Non-Alcoholic Fatty Liver Disease (NAFLD): The role of metabolic syndrome

**DOI:** 10.12669/pjms.41.8.11702

**Published:** 2025-08

**Authors:** Tajwar Gul, Saera Suhail Kidwai, Muhammad Kamran, Hafiz Abdul Basit, Fatima Zahra, Tahir Ansari

**Affiliations:** 1 Tajwar Gul Resident Medicine, Department of Internal Medicine, Fazaia Ruth Pfau Medical College, Karachi, Pakistan; 2 Saera Suhail Kidwai Professor, Department of Internal Medicine, Fazaia Ruth Pfau Medical College, Karachi, Pakistan; 3 Muhammad Kamran Associate Professor, Department of Gastroenterology, Fazaia Ruth Pfau Medical College, Karachi, Pakistan; 4 Hafiz Abdul Basit Assistant Professor, Department of Gastroenterology, Fazaia Ruth Pfau Medical College, Karachi, Pakistan; 5 Fatima Zahra Associate Professor, Department of Internal Medicine, Fazaia Ruth Pfau Medical College, Karachi, Pakistan; 6 Tahir Ansari Professor, Department of Internal Medicine, Fazaia Ruth Pfau Medical College, Karachi, Pakistan

**Keywords:** Fibrosis, Liver, Nonalcoholic fatty liver disease (NAFLD), Metabolic syndrome, Steatosis

## Abstract

**Objective:**

To compare the frequency of degree of hepatic fibrosis and steatosis in patients of Nonalcoholic fatty liver disease (NAFLD) with and without metabolic syndrome.

**Methodology:**

This analytical observational study was carried out at Department of Medicine /gastroenterology FRPMC affiliated hospital from Feb 2024 to July 2024. After ethical approval this study was performed with a sample of 130 patients of Nonalcoholic fatty liver disease (NAFLD) recruited from OPD, randomized into two groups each containing 62 patients: N & M. Group-N patients only had NAFLD and Group-M patients had metabolic syndrome along with NAFLD. Transient Elastography (Fibrotouch) was used to get liver stiffness measurement (LSM) for degree of fibrosis and Ultrasound attenuation parameter (UAP) for degree of steatosis. The primary outcome was frequency of various degrees of fibrosis (F1, F2, F3 &F4) and steatosis (S1, S2, &S3) in both groups.

**Results:**

There were no patients with severe steatosis in Group-N, however, four (7.3%) patients had severe steatosis in Group-M (p value < 0.009). None of the patients had severe fibrosis in Group-N and eight (14.5%) patients had severe fibrosis in Group-M (p value < 0.001).

**Conclusion:**

We concluded that the presence of metabolic syndrome in patients of Nonalcoholic fatty liver disease (NAFLD) is linked to higher degree of steatosis and fibrosis when compared with patients of NAFLD without metabolic syndrome.

## INTRODUCTION

Diabetes and cardiovascular disease are better predicted by a single entity known as metabolic syndrome which is multitude of risk factors leading to diabetes mellitus and hypertension.^1^ Abdominal obesity is the isolated increase in waist circumference of 94 centimeter for men and 80 centimeters for women along with high blood pressure, fasting hyperglycemia or hypertriglyceridemia or decreased high density lipoprotein levels. Metabolic syndrome is an amalgam of these conditions as approved by International Federation for diabetes.^2^

Nonalcoholic fatty liver disease (NAFLD) is signified by liver fat level of greater than 5–10% by weight in absence of underlying liver disease or excessive alcohol use and it is characterized by hepatocyte damage, inflammation and fibrosis.^3^ The pathogenesis involves development of resistance to insulin, derangement of lipid metabolism, metabolic syndrome and increased hepatic fat content and it has been highlighted in different international studies.^4^,^5^ The best indicators of an aggressive disease course of NAFLD is the development of liver fibrosis.^6^ However, a number of other factors are also prevalent in individuals with a severe condition, including diabetes mellitus, advanced age, lipid problems, obesity, and insulin resistance.^7^

These factors have a detrimental effect on the advancement of fibrosis and are crucial in a worsening course of the disease. Diabetes mellitus and hypertriglyceridemia are also connected to increased fibrosis.^8^ Because of the rise in western processed foods and sedentary lifestyles, there is an obesity epidemic that starts in childhood and is accompanied by an increase in diabetes mellitus and nonalcoholic fatty liver disease which is currently the most prevalent form of liver disease with 25% global prevalence.^9^ Similar trends are now seen in Pakistan especially in urban population.^10^,^11^

The rationale of our analytical observational study was to study the frequency of fibrosis in NAFLD with metabolic syndrome and NAFLD without metabolic syndrome in Pakistani population the data of which is scarce, despite of NAFLD’s strong association with MetS and the fact that MetS increases progression to NASH.^12^ Hepatic steatosis and fibrosis are more than three times greater in individuals having metabolic syndrome. More intriguingly metabolic syndrome remains a risk factor for hepatic fibrosis even in absence of steatosis.^13^ Therefore, we aimed to study the frequency of fibrosis in patients with established NAFLD with MetS and those without MetS when measured through non-invasive transient elastography (TE) method. Our study will help in screening and timely intervention of patients who are at risk of development of cirrhosis due to worsening fibrosis and steatosis.

## METHODOLOGY

We carried out our analytical observational study at internal medicine and Gastroenterology department, Fazaia Ruth Pfau Medical College, Karachi. The study continued over a period of six months. We used WHO sample size calculator to find sample size. We used to follow desired unknown parameters: level of significance 10%, power of test 80%, anticipated percentage of patients of NAFLD with metabolic syndrome to develop steatosis to be 26% and anticipated percentage of patients of NAFLD with metabolic syndrome without steatosis to be 74%.^13^ The sample came out to be 13. Similarly, anticipated percentage of patients of NAFLD with metabolic syndrome to develop fibrosis to be 7.5%^13^ and anticipated percentage of patients of NAFLD with metabolic syndrome without fibrosis to be 92.5%.^13^ The minimum sample was 13, keeping 10% significance and 80% power of test. We collected sample through non-probability consecutive sampling on the basis of following inclusion and exclusion criteria.

### Ethical approval:

It was obtained from hospital’s ethical committee (FRPMC-IRB-2023-15 January, 2024.).

### Inclusion criteria:

We included both male and female patients between 20 to 70 years having NAFLD (5-10% hepatic liver fat) diagnosed on ultrasonography. The patients were labeled to have metabolic syndrome if they had at least any three of the following conditions positive: High blood glucose levels (BSF > 7mmol/l, BSR > 11mmol/l) or known diabetic, known Hypertensive on medication or BP greater than 130/90, waist circumference for men to be ≥ 94 cm & for ladies ≥ 80 cm, serum triglycerides ≥150 mg/dl or patient already taking lipid lowering agents for hyperlipidemia, high density lipoprotein (HDL) ≤40 mg/dl for males and ≤50 mg/dl for females or obesity with BMI > 28 Kg/m^2^.^14^

### Exclusion Criteria:

Following patients were excluded: Patients who were positive for hepatitis B and hepatitis C, patients who had history of alcohol abuse and cirrhosis. Patients with ascites, patient with any active pacemaker or defibrillator, active cardiac disease, patients with transplanted liver, patients with hemochromatosis, active malignancy and pregnancy.

The sample was collected through non-probability consecutive sampling and allocated to two groups according to the presence/absence of metabolic syndrome. Group-M included patients who had NAFLD with metabolic syndrome and Group-N included patients who only had NAFLD without any risk factors related to metabolic syndrome. We booked all patients from out-patient department of medicine. We took their consent after carefully explaining the purpose of study. FibroTouch (Wuxi Hisky Medical Technologies Co., Ltd.) was used in all patients to measure fibrosis. Patients were asked to be nil per oral for three hours before TE. Liver stiffness measurement (LSM) and degree of steatosis through ultrasound attenuation parameter (UAP) was measured. A mechanical vibrating probe was positioned vertically between 9^th^ and 11^th^ rib by an experienced operator and ten values were taken while avoiding large vessels utilizing a portion of the liver 6 cm deep and dullest to percussion.

The final number was a computer-generated median value. The FibroTouch device simultaneously measured LSM and UAP.^15^ The primary outcome was frequency of various degrees of steatosis according to Ultrasound Attenuation Parameter (UAP): S1 (Mild steatosis, 240-265DB/M), S2 (Moderate steatosis, 265-295 DB/M) and S3 (Severe steatosis, ≥295 DB/M). Similarly, three categories where fibrosis was defined: F1(Mild fibrosis, LSM 6 to 7 kPa), F2 (moderate fibrosis, LSM 7 TO 9 kPa) F3-F4(severe fibrosis, LSM ≥9 to 12 kPa.

### Statistical Analysis:

The statistical package for social sciences (SPSS) version 26 was employed for statistical analysis. Normality of data was checked by Shapiro-Wilk test. Non-normal data was analyzed by calculating median values and interquartile range and compared through non-parametric tests. Qualitative variables were recorded as frequency and percentages. Quantitative variables were presented as means and standard deviation. Chi-square analysis was employed to compare frequencies. *p* value less than 0.05 was considered significant.

## RESULTS

The total sample size of our study was 130 patients. The primary outcome was frequency of various degrees of fibrosis and steatosis in both groups. Both groups comprised 65 patients of non-alcoholic fatty liver disease (NAFLD). The distribution as per the age and gender is given below in Table-I. Both parameters showing no statistical difference in between the groups however, it can be noted that number of males in both groups is greater than female. The median Body mass index of Group-N was 25.00 Kg/m^2^. The median BMI of Group-M was 30.00 Kg/m^2^ with p value of 0.001. The BMI showed statistical difference. In Group-N patients’ mild steatosis and mild fibrosis was more prevalent as compared to Group-M, however Group-M patients showed more severe degree of steatosis and fibrosis with the p value of less than 0.009 and 0.001 respectively as shown in Table-II. There were no cases of severe fibrosis and steatosis in group-N but there were 4(7.3%) patients with severe steatosis in group-M and 8(14.5%) patients with severe fibrosis in group-M. Chi-square could not be applied in these categories.

**Table-I:** The comparison of demographics of study groups (n=130).

		Group-N n=65	Group-M n=65	P value
Age (Years)		42.00 (IQR 32.50-47.50)	44.00 (IQR 38.00-47.00)	0.438
BMI (Kg/m^2^)		25.00(IQR 24.00-25.48)	30.00(IQR 38.00-47.00)	0.001
		Frequency (%)	Frequency (%)	
Gender	Male	39(60.0)	32(58.2)	0.493
	Female	26(40.0)	23(41.8)	

**Table-II:** The comparison of fibrosis and steatosis of study groups (n=130).

		Group-N n=65 Frequency (%)	Group-M n=65 Frequency (%)	p value
Steatosis	Mild	48(73.8)	30(54.5)	0.009
	moderate	17(26.2)	21(38.2)	
	severe	0(0)	4(7.3)	
Fibrosis	Mild	41(63.1)	31(56.4)	0.001
	moderate	24(36.3)	16(29.1)	
	severe	0(0)	8(14.5)	

## DISCUSSION

The results of our study showed that metabolic syndrome exacerbated fibrosis and steatosis in patients with pre-existing NAFLD. The association between metabolic syndrome and NAFLD has been studied previously by identifying NAFLD with the help of abdominal ultrasound in patients with diabetes and hypertension, however a simple ultrasound does not quantify the degree of steatosis or appreciate if any fibrosis has occurred in NAFLD patients. The degree of steatosis and fibrosis can be identified by using FibroScan (TE)^16^,^17^ however, use of such intervention is not so common in our region of the world and we are coming across many cases of decompensated liver cirrhosis with background of metabolic syndrome. The results of our study implied that metabolic syndrome is leading to worsening and accelerated steatosis and fibrosis in patients of non-alcoholic fatty liver disease (NAFLD) which can be prevented if we scan the patients early, leading to detection of steatosis at an early stage which can be treated and reversed and preventing progression to cirrhosis which is irreversible.

According to Li QQ et al, close monitoring of metabolic syndrome is required in patients who have con-current metabolic syndrome with NAFLD as it accelerates steatosis and fibrosis. Close monitoring will help in timely intervention and disease progression. This is because quite a few Pakistani physicians are oblivious of association as they are more focused on chronic viral hepatitis which is more prevalent in our population.^18^

According to Kani HT et al.^19^ diabetes was the most common factor linked to advanced fibrosis in patients with pre-existing NAFLD, however, they used liver biopsy to confirm patients having NAFLD and we preferred noninvasive ultrasonography to screen patients of NAFLD. They looked into individual risk factors with the fibrosis and they took into account AST as well but we only relied on non-invasive tests.

Halaoui et al.^20^ established the fact that NAFLD is more prevalent among males as it can also be seen in our study. They used fibroscan to identify the degree to fibrosis in males and premenopausal females both younger than 50 years of age and results showed greater degree of fibrosis in males which can be attributed to genetic and hormonal variations among both genders. According to Kaya E et al.^21^ and Vanni E et al.^22^ primary pathogenetic mechanism of NAFLD is insulin resistance which increases free fatty acid synthesis and oxidative stress in liver contributing to hepatic fat accumulation. They advocated that NAFLD is the hepatic manifestation of the metabolic syndrome hence redefined as MAFLD emphasizing metabolic criteria, however it excludes lean NAFLD cases lacking such dysfunction.

Akhtar A et al.^23^ conducted study in tertiary care hospital, Pakistan assessed the correlation between diabetes and NAFLD by comparing concomitant rise in HbA1c levels and alanine aminotransaminase (ALT) level. This finding aligns with the established link between metabolic syndrome and NAFLD prevalence. Similar correlation was found in other studies in Pakistan.^7^ However, the effect of these factors on liver steatosis and fibrosis was not studied.

Arima S et al.^24^ demonstrated in animal study that hypertension may be a risk factor for hepatic fibrosis and liver damage and possible mechanism is glucose intolerance and a reduction in HO-1- or IL-10-mediated anti-inflammatory mechanisms. They demonstrated how a diet deficient of choline affected the extent of hepatic steatosis, liver injury and hepatic fibrosis in spontaneously hypertensive rats. Hypertension is an important component of metabolic syndrome and through this study it has been independently linked to metabolic syndrome. The human studies are also required to develop this independent association. We indirectly linked it by studying metabolic syndrome which incorporates hypertension.

Since steatosis is a diagnostic criterion for NAFLD and fibrosis is linked to a patient’s clinical prognosis, it is crucial to assess both in NAFLD patients. There are numerous tests available for NAFLD patient surveillance. Among these, TE with LSM and UAP measurements are practical and affordable tools for identifying liver steatosis and fibrosis.^25^,^26^ Therefore they can reliably be used for screening of patients. We used TE and our patients were also compliant due it’s non-invasive technique.

However, further areas can also be studied in this regard like interventional studies, assessing the role of lifestyle modification or pharmacological treatment and then monitoring the further progression of fibrosis or steatosis. Impact of individual component of metabolic syndrome on fibrosis can be evaluated separately and then can be compared which component is more contributing toward the fibrosis.

### Strength and Limitation:

Our findings emphasize the importance of identification of steatosis and fibrosis at an early stage in patients having NAFLD by using Fibro Scan as timely management of the disease can mitigate liver damage and improve the outcomes. The study was single Centre. Ultrasonography was used to identify patients of NAFLD which can be a source of bias.

## CONCLUSION

We concluded that the presence of metabolic syndrome in patients of Nonalcoholic fatty liver disease (NAFLD) is linked to higher degree of steatosis and fibrosis underscoring its critical role in disease progression.

**Fig.1:**
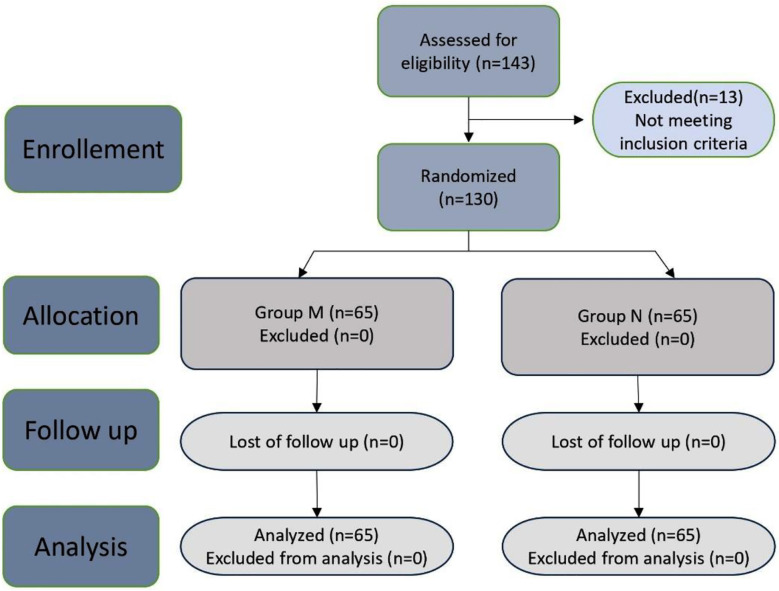
The CONSORT flow diagram of study.

### Authors’ Contributions:

**TG, SSK, MK & HAB:** Drafting of work: design analysis: data acquisition, data interpretation critical analysis of manuscript and accuracy/integrity of work.

**FZ & TA:** Contribution to the conception, data collection, Review/critical analysis and approval of final version to be published. Agreement to be accountable for all aspects of the work
